# Rate Dependent Krasnoselskii-Pokrovskii Modeling and Inverse Compensation Control of Piezoceramic Actuated Stages

**DOI:** 10.3390/s20185062

**Published:** 2020-09-06

**Authors:** Wenjun Li, Linlin Nie, Ying Liu, Miaolei Zhou

**Affiliations:** 1 College of Transportation, Jilin University, Changchun 130022, China; liwj@jlu.edu.cn; 2 College of Communication Engineering, Jilin University, Changchun 130022, China; nielinlinzdh@163.com (L.N.); lying14@mails.jlu.edu.cn (Y.L.)

**Keywords:** hysteresis nonlinearity, inverse compensation, Krasnoselskii–Pokrovskii (KP) model, piezoelectric actuator

## Abstract

The piezoceramic actuated stages have rate-dependent hysteresis nonlinearity, which is not simply related to the current and historical input, but also related to the frequency of the input signal, seriously affects its positioning accuracy. Consider the influence of frequency on hysteresis modeling, a rate-dependent hysteresis nonlinearity model that is based on Krasnoselskii–Pokrovskii (KP) operator is proposed in this paper. A hybrid optimization algorithm of improved particle swarm optimization and cuckoo search is employed in order to identify the density function of rate-dependent KP model, avoiding the blind search process caused by the high randomness of Levy’s flight in the cuckoo search algorithm, and improving the parameter identification performance. For the sake of eliminating the hysteresis characteristics, an inverse feed-forward compensation control that is based on recursive method is proposed without any additional conditions, and a feed-forward compensation controller is designed accordingly. The experimental results show that, under different frequency input signals, as compared with the classic KP model, the proposed rate-dependent KP model can accurately describe the rate-dependent hysteresis characteristics of the piezoceramic actuated stages, and the recursive inverse feed-forward compensation control method can effectively mitigate the hysteresis behaviors.

## 1. Introduction

With the development of technology, precision positioning technology has put forward higher requirements in terms of positioning accuracy and response speed. The piezoceramic actuated stage [1,2,3] is a novel smart material actuator, which is widely applied in micro-nano positioning fields, such as precision engineering, biological engineering, and micro-electronics [4,5]. However, as the core module of the piezoceramic actuated stage, the inherent hysteresis nonlinear characteristic of piezoelectric materials is a problem that cannot be ignored in practical applications.

In recent years, many hysteresis modeling methods have been proposed in order to eliminate the hysteresis nonlinearity of piezoceramic actuated stages. Such models typically have a rate-independent hysteretic nature, which is their output variable does not depend on the first derivative of the input one [6,7,8,9]. Such as Duhem model [10], Bouc–Wen model [11,12], Prandtl–Ishlinskii (PI) model [13,14], Preisach model [15], and Krasnoselskii–Pokrovskii (KP) model [16,17,18]. The Duhem model and Bouc–Wen model are differential equation-based hysteresis models. In [19], in order to describe the asymmetric hysteresis loop, an asymmetric Bouc–Wen model that is based on the recursive least squares online identification method was proposed. The experimental system was established by the limited memory method, and it was verified that this method could effectively improve the modeling accuracy. This kind of differential equation-based hysteresis models have simple construction; however, the modeling accuracy is affected by the relied on the derivative of the input. Preisach model depict hysteresis by superpositing a series of weighted operators. However, the integral operation of Preisach model is difficult to solve [20]. The PI model and KP model are improved from Preisach model. PI model has an accurate analytical inverse, and it has a simpler structure than the Preisach model. However, it still requires a large number of Play operators for modeling which inducing the computational burden during realization. In [21], an entropy-based optimal compression method was proposed to reduce the number of hysteresis operators for a generalized PI model, and the hysteresis behavior of piezoelectric materials was accurately described. In [22], a modified PI model was established to describe a more complex asymmetric hysteresis loop, in which two envelope functions are used instead of threshold *r*. The simulation results verify the effectiveness of characterizing the asymmetric hysteresis nonlinearity. When compared with other operator-based hysteresis models, the KP operator can choose different slope parameters, so that the KP model can describe more varieties of hysteresis nonlinearities. Nevertheless, the KP model requires a lot of mathematical operations in actual application. In order to overcome the computational complexity problem of the KP model, Li et al. [23] introduced a KP model reduction method to acquire the dominant KP kernels, which effectively reduced the computational burden of the KP model while keeping the modeling accuracy. In addition to the above hysteresis models, neural network models [24,25,26,27,28,29,30,31,32], NARMAX models [33,34,35,36,37] and polynomial models [38] are also investigated to describe the hysteresis nonlinearity of piezoelectric and other smart materials.

However, the hysteresis nonlinearity in the piezoelectric materials is not only related to the current and history input, but also related to the frequency of the input signal. The previous models cannot characterize the rate-dependent hysteresis nonlinearity, which seriously affects the positioning accuracy of the piezoceramic actuated stages. In [39], the input function of the classic Play operator was replaced with a dynamic envelope function to enhance the frequency correlation of PI operator, and an improved particle swarm optimization (PSO) algorithm was used to identify the parameters. Subsequently, the rate-dependent hysteresis nonlinearity of piezoelectric ceramics can be described. In [40], a rate-dependent hysteresis modeling method based on fuzzy systems was proposed, and the parameters are optimized by recursive least squares. Xiao et al. [41] presented a modified Preisach inverse model with the superposition of the density function weights, where the density functions and weights were selected by the fast Fourier transform. The proposed rate-dependent inverse model can compensate for the hysteresis characteristics of piezoelectric actuators in different frequency ranges. In [42], an adaptive inverse control strategy based on an improved Elman neural network was proposed. A modified inverse backlash operator was applied as a hidden layer neuron to describe the dynamic behavior of inverse rate-dependent hysteresis to reduce the influence of rate-dependent hysteresis. In [43], the Hammerstein model was proposed in order to describe the rate-dependent hysteresis nonlinearity of piezoceramic actuators. Additionally, the MPI model represented the nonlinear part of the system. On this basis, the control scheme combining PID and inverse model is used for control. A variety of rate-dependent improved models of the previous models have been proposed, however, there seem not many studies on the rate-dependent KP model.

In this paper, a novel rate-dependent KP hysteresis nonlinear model for the piezoceramic actuated stages is proposed. Firstly, a hysteresis model is established while using the KP kernels. Additionally, the frequency factor of the input signal is introduced to obtain the rate-dependent KP (RDKP) hysteresis model. Then, the hybrid optimization algorithm of particle swarm and cuckoo search is used to identify the parameters of density functions in the RDKP model. Finally, inverse feed-forward compensation control based on recursive method is designed for the precision positioning. The experiment was carried out through the piezoceramic actuated stages, and experimental results verify the effectiveness of the proposed modeling and control method.

## 2. Rate-Dependent Hysteresis Modeling of Piezoceramic Actuated Stages

### 2.1. Rate-Dependent Krasnoselskii-Pokrovskii Model

The KP model describes the hysteresis nonlinearity by a weighted superposition of KP operators on the Preisach plane [44]. The KP model presented in [45] is defined as:

(1) $$y\left(t\right)=\int_{p}k_{p}\left[u,\xi_{p}\right]\left(t\right)\mu \left(p\right)dp$$

where $u\left(t\right)$ and $y\left(t\right)$ are the input voltage and output displacement of system, respectively. $k_{p}\left[u,\xi_{p}\right]\left(t\right)$ is a single KP kernel, $\mu \left(p\right)$ denotes the density distribution function of Preisach plane *p*, where $p=\left\{p\left(p_{1},p_{2}\right)\in R^{2}\left.\right|0\leq p_{1}\leq p_{2}\leq 1\right\}$, $\xi_{p}$ is the memory term which is updated when $\dot{u}\left(t\right)$ changes its sign.

**Figure 1:**
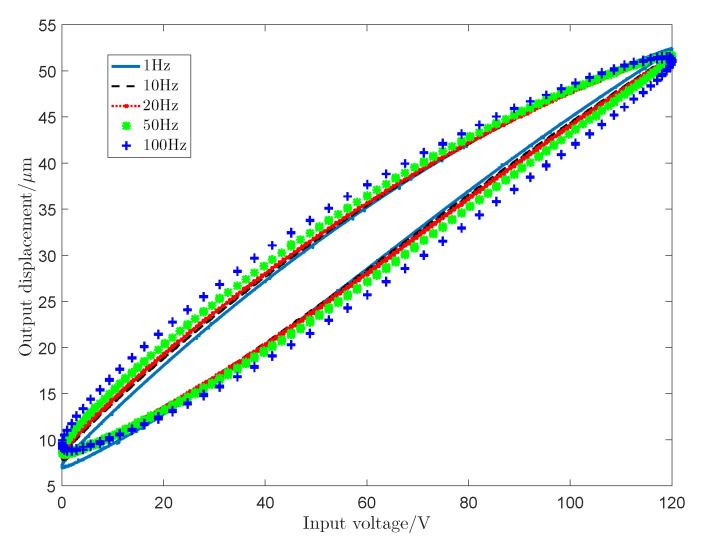
Hysteresis loops under different input frequency.

It can be seen from Figure 1 that the piezoceramic actuated stages have a rate-dependent characteristic in hysteresis nonlinearity. The classic KP model is a static hysteresis model, which cannot precisely characterize the rate-dependent hysteresis. Therefore, based on the classic KP model, the frequency factor of input signal is introduced into the hysteresis model to improve the rate-dependent characteristics. Subsequentlys, a kind of RDKP hysteresis nonlinear model is established as:

(2) $$y\left(t\right)=\int_{p}k_{p}\left[u,\xi_{p}\right]\left(t\right)\mu \left(p\right)\psi \left(\dot{u}\left(t\right)\right)dp$$

The added input voltage change rate function is expressed as:

(3) $$\psi \left(\dot{u}\left(t\right)\right)=a_{1}e^{-a_{2}\dot{u}\left(t\right)}$$

where $a_{1}$, $a_{2}$ are positive constant values. It can be seen from Figure 1 that, as the frequency of the input voltage signal increases, the maximum output displacement becomes smaller. $a_{1}$ in (3) is used to describe this characteristic; as the frequency of the input voltage signal increases, the hysteresis loop becomes wider, that is, the value of the rising loop becomes smaller, and the value of the falling loop becomes larger. $e^{-a_{2}\dot{u}\left(t\right)}$ in (3) is used to describe this characteristic. When the input voltage gradually increases, $\dot{u}\left(t\right)>0$, and $e^{-a_{2}\dot{u}\left(t\right)}\in \left(0,1\right)$. Afterwards, multiplied by the previous output value and the final value decreases; when the input voltage signal gradually decreases, $\dot{u}\left(t\right)\leq 0$ and $e^{-a_{2}\dot{u}\left(t\right)}\geq 1$, so multiplying with the previous value, the final value becomes larger.

**Figure 2:**
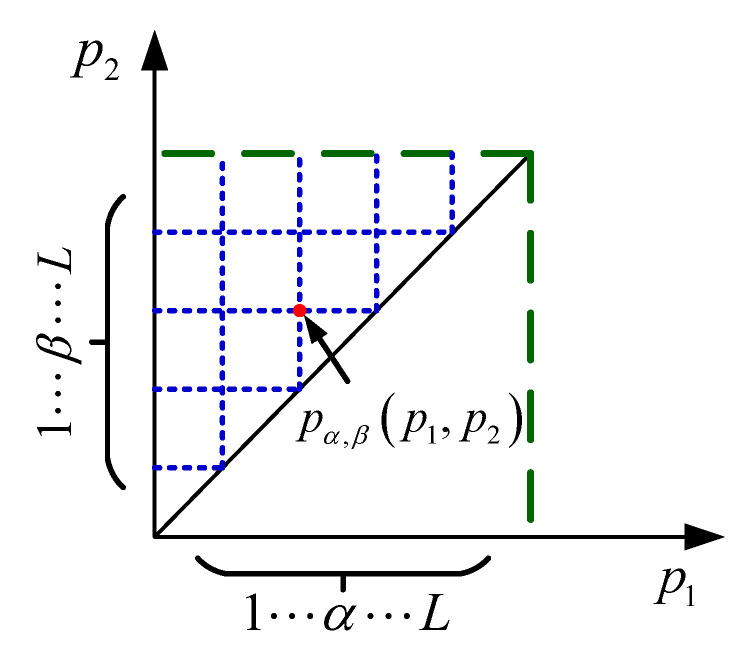
Discrete Preisach plane.

The KP operator, which is shown in Figure 2, is given by the following equation:

(4) $$k_{p}\left[u,\xi_{p}\right]\left(t\right)=\left\{\begin{array}{c} \max\left\{\xi_{p}\left(t\right),s\left(u\left(t\right)-p_{2}\right)\right\}\dot{u}>0\\ \min\left\{\xi_{p}\left(t\right),s\left(u\left(t\right)-p_{1}\right)\right\}\dot{u}\leq 0 \end{array}\right.$$

The memory term $\xi_{p}$ can be expressed as:

(5) $$\xi_{p}\left(t\right)=\left\{\begin{array}{cc} 0 & if\;t=t_{0}\\ k_{p}\left[u,\xi_{p}\left(t_{m-1}\right)\right]\left(t\right) & if\;t=t_{m}>t_{m-1}\;and\;sign\left(\dot{u}{(t}^{+})\right)=-sign\left(\dot{u}\left(t^{\_}\right)\right)\\ \xi_{p}\left(t_{m-1}\right) & if\;t_{m}>t>t_{m-1}\;and\;sign\left(\dot{u}{(t}^{+})\right)=sign\left(\dot{u}\left(t^{\_}\right)\right) \end{array}\right.$$

where *m* is the number of sign changes of $\dot{u}\left(t\right)$, $\dot{u}\left(t^{+}\right)$ represents the first moment after $\dot{u}$ change its sign and $\dot{u}\left(t^{-}\right)$ represents the last moment before $\dot{u}$ change its sign. s(u(t)) is boundary function of KP operators, which can be expressed as:

(6) $$s\left(u\left(t\right)\right)=\left\{\begin{array}{ccc} 0 & if & u\left(t\right)<0\\ u\left(t\right)/a & if & 0\leq u\left(t\right)\leq a\\ 1 & if & u\left(t\right)>a \end{array}\right.$$

where $a=\frac{1}{L-1}$.

**Figure 3:**
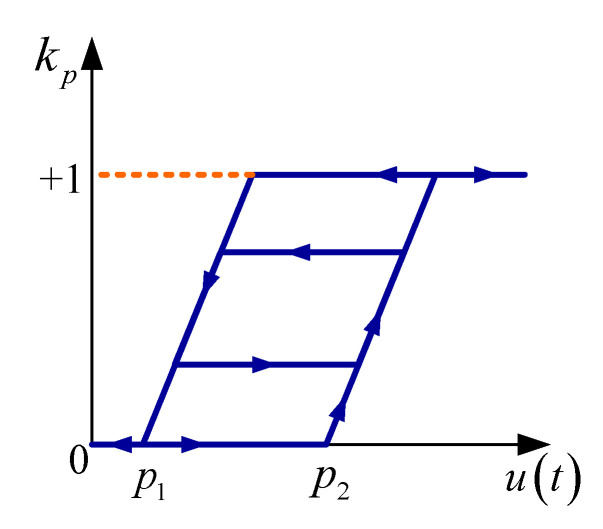
KP hysteresis operator.

The integral form of Equation (2) is not easy to calculate. In order to facilitate the realization of the RDKP model, divide the Preisach plane into L×L parts with N=(L+1)(L+2)/2 cells, like Figure 2, every point $p_{\alpha ,\beta }\left(p_{1},p_{2}\right)$ represents a KP operator. When L→∞, the discrete Preisach plane is approximately a continuous Preisach plane. As shown in Figure 2 and Figure 3, each discrete KP operator corresponds to a density parameter $\mu \left(p_{i},p_{j}\right)\left(i=1,2,\cdots ,L,j=i=1,2,\cdots ,i\right)$ on the discrete Preisach plane. Subsequently, a discrete RDKP model expression is given by the discrete and superposition principle:

(7) $$y\left(t\right)=\sum_{i=1}^{L}\sum_{j=1}^{i}k_{p}\left[u,\xi_{p}\right]\left(t\right)\mu \left(p_{i},p_{j}\right)a_{1}e^{-a_{2}\dot{u}\left(t\right)}$$

From (7), the output displacement of the RDKP model is the superposition of the products of each KP operator and the corresponding density function. The KP operator can be calculated by Equations (4)–(6), and the density parameters $\mu \left(p_{i},p_{j}\right)$ in (7) can be identified by the hybrid optimization algorithm of particle swarm and cuckoo, which will be introduced in the next part.

### 2.2. Identification of the Density Function by Hybrid Optimization Algorithm of Particle Swarm and Cuckoo Search

When compared with the other intelligent optimization algorithms, the cuckoo search (CS) algorithm has fewer parameters, and it is not easy to fall into the local optimum. However, in the iterative update process of the CS algorithm, the high randomness of Levy’s flight leads to a more blind search process and slower convergence speed, especially when approaching the optimal solution. Therefore, a hybrid optimization algorithm of Particle Swarm Optimization and Cuckoo Search (PSO-CS) [46,47] is proposed to identify the density parameters of the RDKP model in order to improve the search performance of the CS algorithm.

Firstly, the PSO algorithm is used to update the speed and position of the particles:

(8) $$\begin{array}{cc} V_{i}^{k+1} & =\omega V_{i}^{k}+c_{1}r_{1}\left[p_{ibest}^{k}-X_{i}^{k}\right]+c_{2}r_{2}\left[p_{gbest}^{k}-X_{i}^{k}\right] \end{array}$$

(9) $$\begin{array}{cc} X_{i}^{k+1} & =X_{i}^{k}+V_{i}^{k+1} \end{array}$$

where $X_{i}^{k}=\left(x_{i1},x_{i2},...,x_{im}\right)$ and $X_{i}^{k+1}=\left(x_{i1},x_{i2},...,x_{im}\right)$ are the position of the *k*th and k+1th generation of the *i*th particle in the m—dimensional search space, respectively; $V_{i}^{k}=\left(v_{i1},v_{i2},...,v_{im}\right)$ and $V_{i}^{k+1}=\left(v_{i1},v_{i2},...,v_{im}\right)$ are the speed of the *k*th and k+1th generation of the *i*th particle in the m—dimensional search space, respectively; $p_{ibest}^{k}=\left(p_{i1},p_{i2},...,p_{im}\right)$ is the optimal position of each particle in the *k*th generation; $p_{gbest}^{k}=\left(p_{g1},p_{g2},...,p_{gm}\right)$ is the global optimal position; ω is the inertia weight value, which controls the variation range of particle speed; $c_{1},c_{2}$ are acceleration constants, which represent the random acceleration weights of particles approaching their own extreme value and global extreme value, usually taking a value of 2; $r_{1},r_{2}$ are uniformly distributed random numbers between [0,1]. In addition, in order to avoid particles flying away from the search space during the search process, the speed range of each particle is usually set to $\left[v_{min},v_{max}\right]$ and the position range to $\left[x_{min},x_{max}\right]$. In general, ω in (9) decreases linearly from the maximum weight $\omega_{max}$ to the minimum weight $\omega_{min}$, namely:

(10) $$\omega \left(k\right)=\omega_{\max}-\frac{\omega_{\max}-\omega_{\min}}{T_{\max}}k$$

where *k* is the number of contemporary iterations; $T_{max}$ is the total number of iterations.

Subsequently, keep the global optimal position $p_{gbest}$, and substitute the optimal position $p_{ibest}$ of the particle into the CS algorithm, and continue to iterative update, the update formula is:

(11) $$p_{ibest}^{k+1}=p_{ibest}^{k}+\alpha_{0}\frac{\varphi \times u}{{\left|v\right|}^{\frac{1}{\lambda }}}\left(p_{ibest}^{k}-p_{gbest}^{k}\right)$$

where $a_{0}$ is a constant, *u* and *v* follow the standard normal distribution; λ=1.5; the expression of φ is:

(12) $$\varphi ={\left\{\frac{\Gamma \left(1+\lambda \right)\times \sin\left(\pi \times \frac{\lambda }{2}\right)}{\Gamma \left\{\left[\frac{1+\lambda }{2}\right]\times \lambda \times 2^{\frac{\lambda -1}{2}}\right\}}\right\}}^{\frac{1}{\lambda }}$$

**Figure 4:**
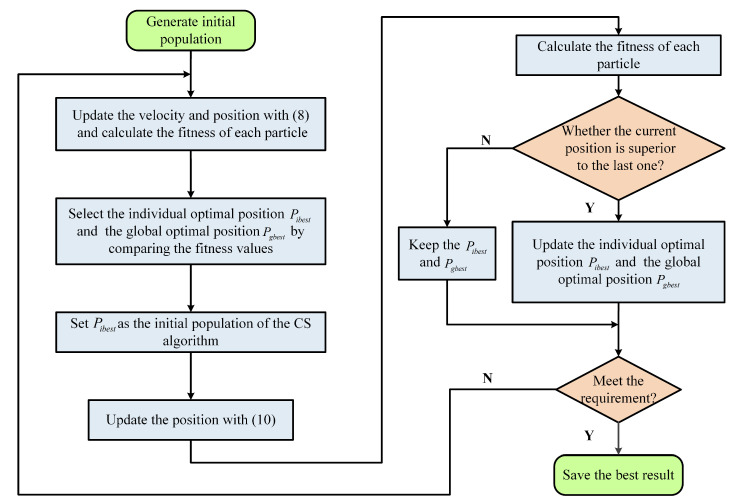
Flow chart of Particle Swarm Optimization and Cuckoo Search (PSO-CS) hybrid optimization algorithm.

Figure 4 shows the specific process of the PSO-CS algorithm:

## 3. Inverse Feed-Forward Compensation Control Based on Recursive Method

**Figure 5:**
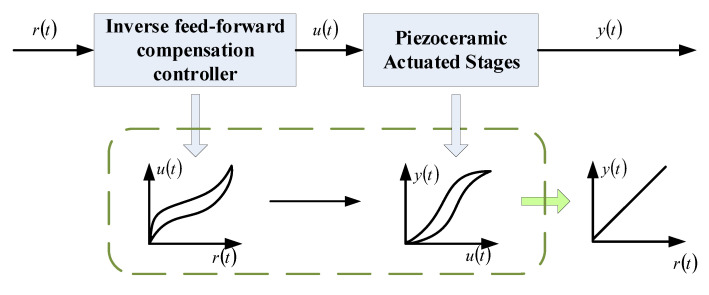
The principle of inverse feed-forward compensation control.

Designing an inverse feed-forward compensation controller is an effective method to eliminate the effects of hysteresis behavior. It compensates the hysteresis nonlinearity by establishing the inverse model of the controlled system based on the invariance principle and its extension. The inverse feed-forward compensation control principle is as shown in Figure 5.

At present, the feed-forward compensation controller is generally designed by direct deduction of formula, which is, deriving its inverse model according to the formulas of positive RDKP model directly. This method requires a good understanding of the structure of the hysteresis nonlinear model, and the hysteresis part needs a separate expression. Many hysteresis nonlinear models have no conditions to derive the inverse model directly.

Therefore, in this paper, when considering the structure of the RDKP model, the recursive inverse feed-forward compensation (RIFC) control is proposed. The recursive inverse feed-forward compensator uses a stepwise recursive approach to solve the inverse model. This method does not require any additional conditions and is suitable for all inverse nonlinear hysteresis models. The core idea of the RIFC method is to obtain the ideal output voltage $u\left(t\right)$ of the RIFC controller at time *t* according to the input voltage value $u\left(t-1\right)$ (denoted as “$u_{dpresent}$”), output displacement value $y\left(t-1\right)$ (denoted as “$y_{dpresent}$”), the desired output displacement signal $r\left(t\right)$, and the hysteresis model. Firstly, compare the desired output displacement *r* with the model output displacement $y_{dpresent}$. If $r>y_{dpresent}$, the system output *y* is in an increasing state. It means that the input voltage *u* is increasing. So, we add the Δu to input voltage $u_{dpresent}$, and substitute the updated input voltage $u_{dpresent}$ into the RDKP model (7) to calculate the corresponding output displacement $y_{dpresent}$. Compare *r* and $y_{dpresent}$ again, if *r* is still greater than $y_{dpresent}$, continue to increase the input voltage until *r* is less than $y_{dpresent}$ for the first time. By this time, this $u_{dpresent}$ is the required input voltage. It is similar when the system output *y* is in a decreasing state. The control rules of the RIFC controller are as follows:

Step 1. Set $u_{d}=u_{dpresent}$ and $y_{d}=y_{dpresent}$ ( $u_{d}$ and $y_{d}$ are two intermediate variables defined).

**Figure 6:**
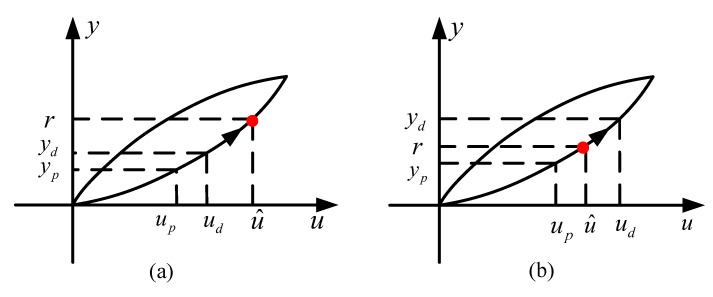
The change of desired output displacement as system input increases, including (**a**) the current output displacement is less than the desired output displacement and (**b**) the current output displacement exceeds the desired output displacement for the first time.

Step 2. If $r>y_{dpresent}$, which is, the desired output displacement is greater than the current output displacement. The system is in an increasing state, as shown in Figure 6a.

Step 2.1 Set $u_{p}=u_{d}$ and $y_{p}=y_{d}$ ( $u_{p}$ and $y_{p}$ are also two intermediate variables defined).

Step 2.2 Update $u_{d}$ as $u_{d}=u_{p}+\Delta u$, where Δu is the iteration step size. Additionally, then update $y_{d}$ using RDKP model (7).

Step 2.3 If $r>y_{d}$, turn back to step 2.1 and continue, the input voltage u(t) should be added until the output displacement $y_{d}$ equal to or more than the desired displacement r(t).

Step 2.4 When $y_{d}$ satisfies $y_{d}>r$ firstly, as shown in Figure 6b, update the required input voltage signal $\hat{u}$, as:

(13) $$\hat{u}=u_{p}+\frac{r-y_{p}}{y_{d}-y_{p}}\Delta u$$

**Figure 7:**
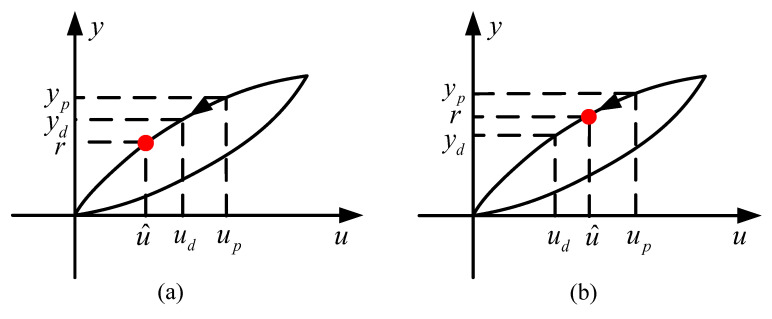
The change of desired output displacement as system input decreases, including (**a**) the current output displacement is greater than the desired output displacement and (**b**) the current output displacement is less than the desired output displacement for the first time.

Step 3. If $r<y_{dpresent}$, which is, the desired output displacement is less than the system output displacement in the current moment. The system is in a decreasing state, as shown in Figure 7a.

Step 3.1 Set $u_{p}=u_{d}$ and $y_{p}=y_{d}$.

Step 3.2 Update $u_{d}$ as $u_{d}=u_{p}-\Delta u$, and update $y_{d}$ using RDKP model (7).

Step 3.3 If $r<y_{d}$, turn back to step 3.1 and continue, the input voltage u(t) should be reduced until the output displacement $y_{d}$ equal to or less than desired displacement r(t).

Step 3.4 When $y_{d}$ satisfies $y_{d}<r$ firstly, as shown in Figure 7b, update the required input voltage signal $\hat{u}$ as:

(14) $$\hat{u}=u_{p}-\frac{r-y_{p}}{y_{d}-y_{p}}\Delta u$$

Suppose that the actual input voltage is $u^{*}\left(t\right)$ when the desired output displacement is r(t). According to the above recursive algorithm, it is easy to obtain that $u_{p}\leq \hat{u}\leq_{d}$ and $u_{p}\leq u^{*}\leq u_{d}$. Subsequently, we get $\left|u^{*}-\hat{u}\right|\leq \Delta u$. That is, the estimation error solved by the recursive algorithm does not exceed Δu. If Δu→0, then $\hat{u}\to u^{*}$. It can be seen that the smaller the value of Δu, the more accurate the value of the estimated input voltage. However, when the Δu is smaller, the computational burden of the RIFC controller is greater. In this paper, the appropriate u(t) is chosen through repeated numerical simulation.

## 4. Experimental Results

In this section, the proposed modeling approach and control strategy are tested on piezoceramic actuated stages to evaluate its efficiency.

The experimental device consists of a piezoceramic actuated stage (MPT-2MRL102A), a host computer, a data acquisition card (PCI-1710), and an integrated positioning controller. The piezoceramic actuated stage with a displacement sensor of strain foil can reach 60μm maximum output displacement.

**Figure 8:**
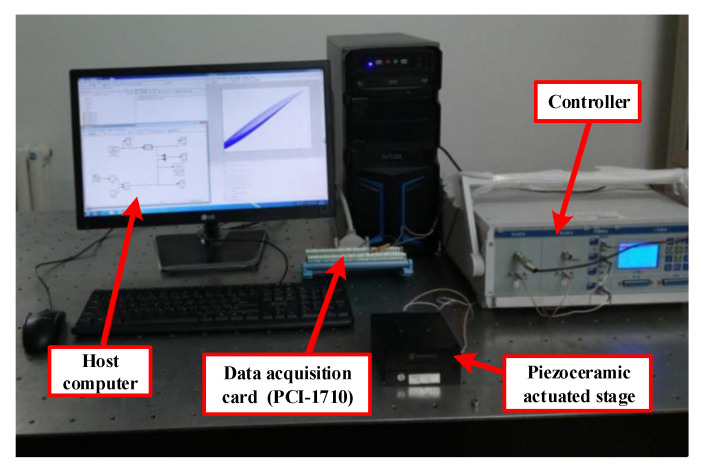
Experimental environment of piezoceramic actuated stage control system.

**Figure 9:**
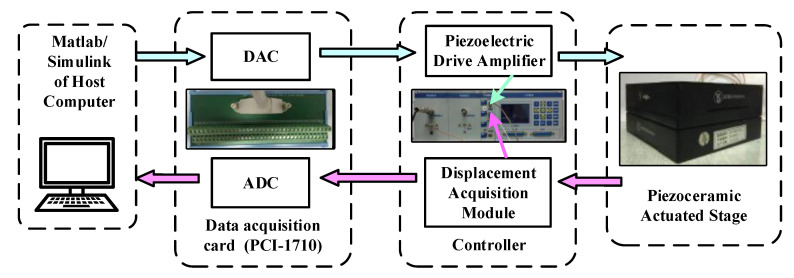
The experimental device structure of piezoceramic actuated stage control system.

The real-time control signal is generated by RTW of Matlab/Simulink and translates into an analog voltage signal after via a 16-bit data acquisition card. The signal is enlarged by piezoelectric drive amplifier and it actuates the piezo-actuated stage motion. Subsequently, the displacement signal that is measured by the sensor is transformed into the host computer the via data acquisition card. The experimental environment and experimental device structure of piezoceramic actuated stage control system are shown in Figure 8 and Figure 9.

### 4.1. The Experimental Results of the Rate-Dependent Kp Model

In this part, in order to verify the accuracy of the proposed RDKP model, sinusoidal signal at various frequency and sinusoidal signal with decreasing amplitude are used to excite the piezoceramic actuated stages. Meanwhile, the rate-independent classic KP model is used as a comparison. The sinusoidal input voltage signal is selected as $u\left(t\right)=60\sin\left(2\pi *ft+3/2\pi \right)+60$, where *f* is the frequency of the input voltage signal selected as 1 Hz, 10 Hz, 20 Hz, 50 Hz, and 100 Hz, respectively. Additionally, the parameters $a_{1}$ and $a_{2}$ are selected as 0.96 and $2.5\times 10^{7}$, respectively.

**Figure 10:**
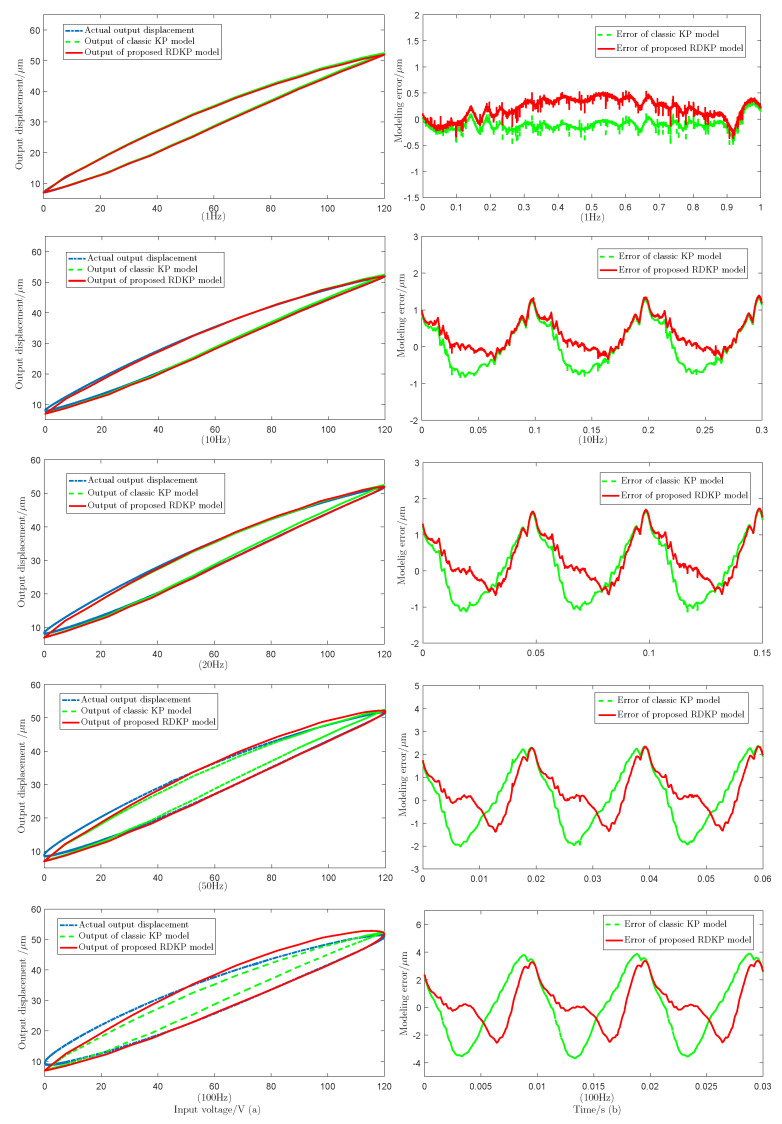
The experiment results of the proposed rate-dependent Krasnoselskii–Pokrovskii (RDKP) model and classic KP model with diverse frequency input voltage, including (**a**) hysteresis loops of input voltage and output displacements and (**b**) modeling errors of the proposed RDKP model and classic KP model.

**Figure 11:**
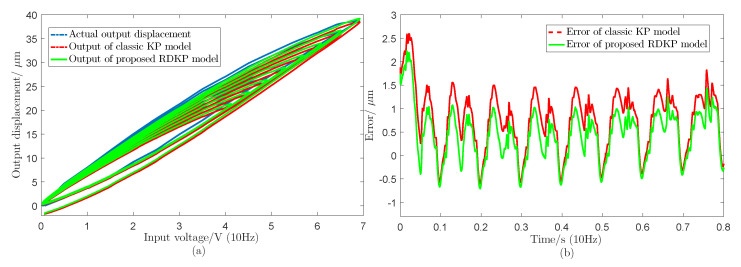
The experiment results of the proposed RDKP model and classic KP model under sinusoidal signal with damping amplitude input voltage, including (**a**) minor hysteresis loops of input voltage and output displacements and (**b**) modeling errors of the proposed RDKP model and classic KP model.

Figure 10 shows the modeling performance of the proposed RDKP model and the classic KP model with 1 Hz, 10 Hz, 20 Hz, 50 Hz, and 100 Hz input voltage signal, respectively. Table 1 shows the root-mean-square error (RMSE) values and the relative error (RE) values of the actual output and model output with different input voltage signal frequency. Additionally, Figure 11 shows the modeling performance under sinusoidal input voltage signal with decreasing amplitude.

It can be seen from Figure 10 that, at the same frequency, the tracking error of the proposed RDKP model is smaller than the rate-independent classic KP model, except when the input signal frequency is 1 Hz. This can be explained by the fact that the density parameters are identified by the classic KP model with the input signal frequency is 1 Hz. When the input voltage signal frequency is 10 Hz, 20 Hz, 50 Hz, and 100 Hz, the RMSE is reduced by 9.08%, 13.49%, 24.23%, and 33.20%, respectively. Therefore, it can be concluded that, as the frequency increases, the approximation effect of the RDKP model becomes increasingly obvious, which is, the approximate error of the classic KP model increases more, while the tracking error of the RDKP model increases less. Through the error curves that are shown in Figure 11b, it can be calculated that the RMSE and RE of the classic KP model are 1.0076 μm and 5.57%, respectively. Additionally, the RMSE and the RE of the proposed RDKP model are 0.7065 μm and 3.83%, respectively. The experimental results proved that the proposed RDKP model can effectively describe the rate-dependent hysteresis behavior of the piezoceramic actuated stages when the input voltage signals are sinusoidal signal and decreasing amplitude signal.

### 4.2. The Experimental Results of the Recursive Inverse Feed-Forward Compensation Control

Different forms of reference displacement signals are selected to conduct displacement tracking and control experiments based on RDKP model and classic KP model in order to confirm the effectiveness of RIFC control strategy.

#### 4.2.1. Displacement Tracking Control under the Sinusoidal Reference Signals

The desired trajectory is selected as $r\left(t\right)=18\sin\left(2\pi *ft+3/2\pi \right)+28$, where *f* is the frequency of the input voltage signal selected as 1 Hz, 10 Hz, 20 Hz, 50 Hz, and 100 Hz, respectively. In addition, the parameter Δu of RIFC control is selected as 0.01.

**Figure 12:**
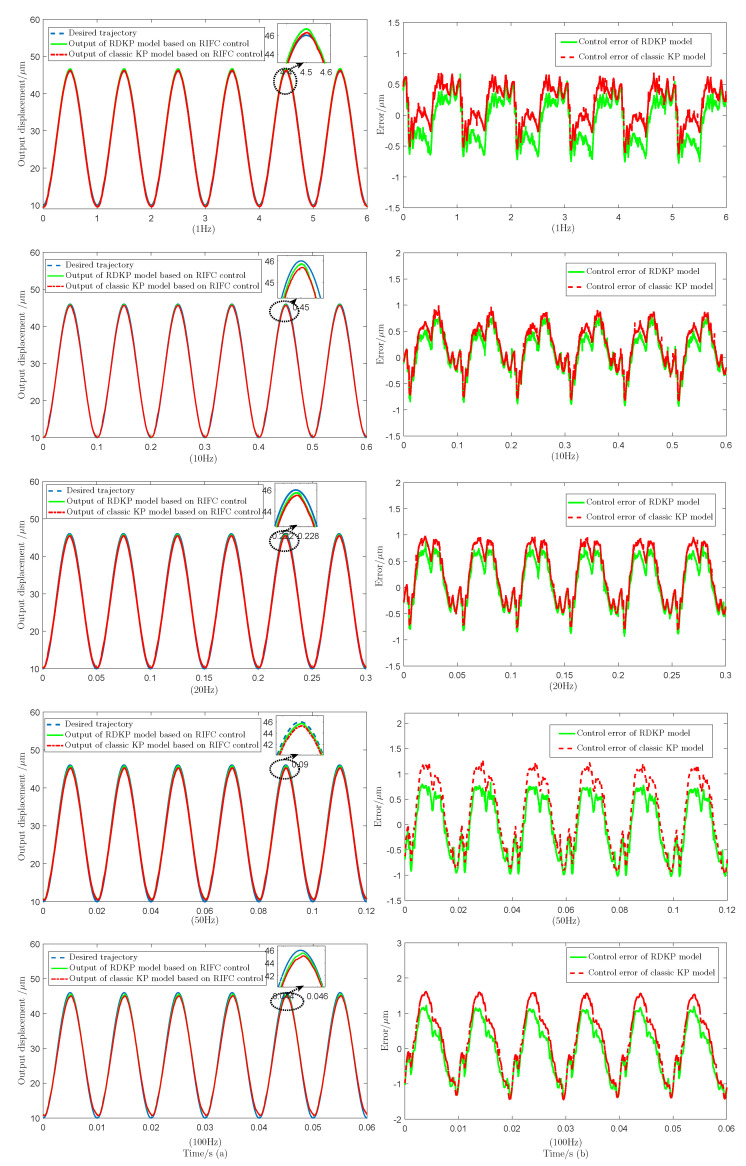
The tracking performance of the RDKP model and classic KP model based on recursive inverse feed-forward compensation (RIFC) control with diverse input voltage frequency, including (**a**) the desired and control output tracking displacements (**b**) control errors of the proposed RDKP model and classic KP model.

Figure 12 shows the tracking performance of the RIFC control that is based on the RDKP model and classic KP model with 1 Hz, 10 Hz, 20 Hz, 50 Hz, and 100 Hz input voltage signal, respectively. Table 2 shows the tracking RMSE values and the tracking RE values of the proposed RDKP model and the classic KP model with different input voltage signal frequency.

The experimental results indicate that, at different frequency, the proposed RIFC control greatly reduces the effect of hysteresis nonlinearity. Additionally, at the same frequency, except when the input signal frequency is 1 Hz, the proposed RDKP model has a smaller positioning error and a higher accuracy than the classic KP model based on RIFC control. When the input voltage signal frequency is 10 Hz, 20 Hz, 50 Hz, and 100 Hz, the RMSE is reduced by 0.0598, 0.1008, 0.1569, and 0.1759, respectively. As the frequency increase, the control effect becomes increasingly obvious. It can be seen that the reduction of the tracking error value gradually increases. The effectiveness and feasibility of the RIFC control are verified.

#### 4.2.2. Displacement Tracking Control under the Triangular Reference Signals

The desired trajectory is selected as a triangular wave signal with the amplitude of 40 μm, and the frequency of the input voltage signal is selected as 1 Hz, 10 Hz, 20 Hz, 50 Hz, and 100 Hz, respectively. The parameter Δu of RIFC control is still selected as 0.01.

**Figure 13:**
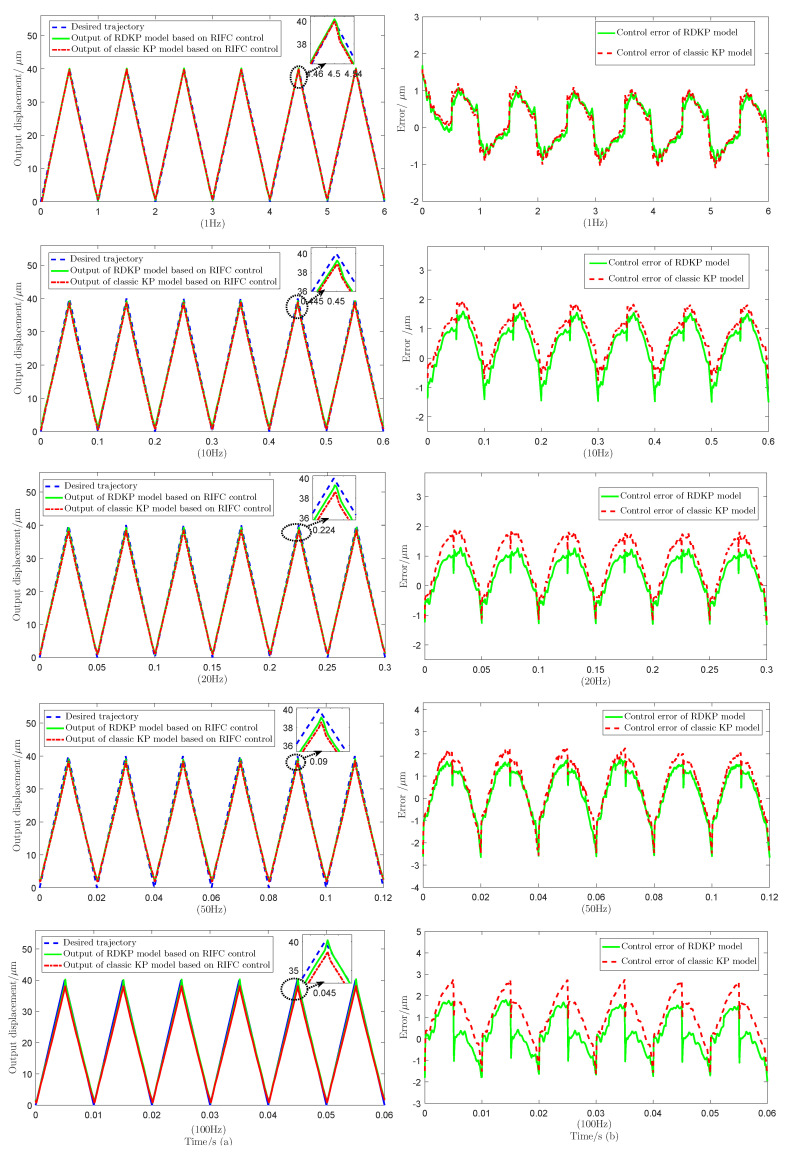
The tracking performance of the RDKP model and classic KP model based on RIFC control with diverse input voltage frequency, including (**a**) the desired and control output tracking displacements (**b**) control errors of the proposed RDKP model and classic KP model.

Figure 13 shows the tracking performance of the RIFC control based on RDKP model and classic KP model under triangular reference signals. Table 3 shows the tracking RMSE values and the tracking RE values of the proposed RDKP model and the classic KP model with different input voltage signal frequency.

Figure 13 indicates that, at different frequency, the proposed RIFC control greatly reduces the effect of hysteresis nonlinearity. Additionally, at the same frequency, the proposed RDKP model has a smaller tracking error and higher accuracy than the classic KP model that is based on RIFC control. It can be seen from Table 3 that, as the input voltage signal frequency increases, the RMSE is reduced by 0.0472, 0.1763, 0.3767, 0.2386, and 0.5468, respectively. The effectiveness and feasibility of the RIFC control are verified.

## 5. Conclusions

In this paper, a rate-dependent hysteresis nonlinearity modeling and a trajectory tracking control method of piezoceramic actuated stages are studied. A RDKP hysteresis nonlinear model based on the rate-dependent KP kernel is developed in order to describe the hysteresis behavior of the piezoceramic actuated stages under different frequency. Additionally, the PSO-CS optimization algorithm, a hybrid optimization algorithm of particle swarm optimization and cuckoo search, is adopted in order to obtain the accurate RDKP model parameters. By comparing with the real measured data from the piezoceramic actuated stages, the identified RDKP model can describe the actual hysteresis loop more accurately than the traditional KP model, and the higher the frequency of the input signal, the better the description of the RDKP model. In order to eliminate the influence of hysteresis behavior, a RIFC control based on a recursive method is proposed. The experimental results indicate that the proposed RIFC control method can effectively reduce the influence of hysteresis nonlinear behavior on positioning precision.

## Figures and Tables

**Table 1:** Comparison of modeling errors between the classic KP model and the proposed RDKP model with diverse input frequency.

Input Frequency	Model	RMSE (μm)	RE (%)
1 Hz	KP model	0.1482	0.42
	RDKP model	0.2761	0.80
10 Hz	KP model	0.5997	1.72
	RDKP model	0.5452	1.57
20 Hz	KP model	0.8345	2.39
	RDKP model	0.7219	2.08
50 Hz	KP model	1.3916	3.89
	RDKP model	1.0543	3.04
100 Hz	KP model	2.4464	7.01
	RDKP model	1.6341	4.69

**Table 2:** Comparison of tracking errors between the classic KP model and the proposed RDKP model under sinusoidal trajectories with diverse input frequency based on the RIFC control method.

Input Frequency	RIFC Control Based on KP Model (RMSE (μm) / RE (%))	RIFC Control Based on RDKP Model (RMSE (μm) / RE (%))
1 Hz	0.3435 / 1.12	0.3649 / 1.18
10 Hz	0.4626 / 1.52	0.4028 / 1.30
20 Hz	0.5734 / 1.89	0.4726 / 1.54
50 Hz	0.7379 / 2.44	0.5810 / 1.90
100 Hz	0.9416 / 3.08	0.7657 / 2.51

**Table 3:** Comparison of tracking errors between the classic KP model and the proposed RDKP model under triangular trajectories with diverse input frequency based on the RIFC control method.

Input Frequency	RIFC Control Based on KP Model (RMSE (μm) / RE (%))	RIFC Control Based on RDKP Model (RMSE (μm) / RE (%))
1 Hz	0.6325 / 2.77	0.5853 / 1.18
10 Hz	1.0625 / 4.81	0.8862 / 3.96
20 Hz	1.1145 / 5.06	0.7378 / 3.29
50 Hz	1.3261 / 6.03	1.0875 / 4.87
100 Hz	1.4649 / 6.75	0.9181 / 4.07
